# Unilateral J‐cut division versus partial and subtotal removal techniques in female patients with mesh‐related urethral obstruction: Multicentric comparative study

**DOI:** 10.1002/bco2.350

**Published:** 2024-03-25

**Authors:** Bülent Çetinel, Göktuğ Kalender, Elif Altınay Kırlı, Aydın Yenilmez, Ömer Gülpınar, Adnan Şimşir, Gökhan Temeltaş, Alkan Çubuk, Günay Can

**Affiliations:** ^1^ Cerrahpaşa Faculty of Medicine, Department of Urology Istanbul University‐Cerrahpaşa Istanbul Turkey; ^2^ Faculty of Medicine, Department of Urology Eskişehir Osmangazi University Eskişehir Turkey; ^3^ Faculty of Medicine, Department of Urology Ankara University Ankara Turkey; ^4^ Faculty of Medicine, Department of Urology Ege University Bornova Turkey; ^5^ Faculty of Medicine, Department of Urology Celal Bayar University Manisa Turkey; ^6^ Faculty of Medicine, Department of Urology Kırklareli University Kırklareli Turkey; ^7^ Cerrahpaşa Faculty of Medicine, Department of Public Health Istanbul University‐Cerrahpaşa Istanbul Turkey

**Keywords:** bladder outlet obstruction, de‐novo stress urinary incontinence, mesh‐related obstruction, sling excision, sling incision, surgical outcomes, voiding dysfunction

## Abstract

**Objective:**

To compare the functional (obstruction relieving) outcomes and complications of unilateral J‐cut division, partial and subtotal vaginal removal techniques were performed for mesh‐related urethral obstruction (MRUO) in females.

**Methods:**

Patient review included demographics, a medical history and proforma with details of lower urinary tract symptoms (LUTS), physical and urodynamic findings, detailed surgical reports and follow‐up data. Variables were compared between the three groups.

**Results:**

Out of 130 patients with sling revision surgery (SRS), 54 women underwent SRS for MRUO with a median follow‐up of 48 (17–96) months. Unilateral J‐cut division, partial and subtotal vaginal removal techniques were performed in 12, 31 and 11 patients with a median duration of surgery of 30 (25–34), 40 (35–56) and 60 (60–70) minutes, respectively (*p* = 0.001). Statistically significant increase in median maximum free urine flow rate and decrease in median post‐void residual urine volume were found after SRS in the three groups, while de novo stress urinary incontinence (SUI) developed in 10%, 44% and 60% of the patients in the unilateral J‐cut division, partial and subtotal removal groups, respectively (*p* = 0.007).

**Conclusions:**

The unilateral J‐cut division technique was as effective as the partial and subtotal vaginal removal techniques in relieving MRUO with a shorter duration of surgery time (*p* = 0.001) and lower risk of de novo SUI (*p* = 0.007). Comparative studies with a larger number of patients are needed.

## 1 INTRODUCTION

Synthetic mid‐urethral slings are the gold standard treatment for female stress urinary incontinence (SUI).^1^ This type of surgery may have early and late complications such as mesh exposure, recurrent urinary tract infection, persistent or de novo urgency/SUI, transient or persistent urethral obstruction, pain and dyspareunia, and some of these complications can be significantly troublesome.^2^, ^3^, ^4^

Sling revision surgery (SRS) for mesh‐related complications consists of the division, partial, subtotal or total removal of the mesh by vaginal and/or abdominal (open or laparoscopic/robotic) approaches.^4^, ^5^, ^6^, ^7^, ^8^, ^9^, ^10^, ^11^, ^12^, ^13^, ^14^

There is a tendency to perform the division technique in patients with mesh‐related urethral obstruction (MRUO), and the partial, subtotal and total removal techniques for pain, exposure and extrusion complications. The partial, subtotal and even total removal techniques are still being used to treat MRUO.^6^, ^7^, ^8^, ^12^, ^13^, ^14^, ^15^, ^16^, ^17^, ^18^, ^19^ Evidence from comparative studies is needed to provide recommendations for the choice of the ideal surgical technique in MRUO.

The division technique for MRUO can be performed midline or laterally. Kasturi et al. described the unilateral J‐cut technique in 15 patients with MRUO. The authors stated that sling division in the midline might compromise support under the urethra and potentially increase the risk of SUI recurrence.^14^ According to our knowledge, no study to date compared the unilateral J‐cut technique with the partial and subtotal vaginal removal techniques in patients with MRUO based on functional (obstruction relieving) outcomes and complications.

This study aimed to compare the functional (obstruction relieving) outcomes, complications (recurrence of SUI) and duration of surgery of the unilateral J‐cut division, partial and subtotal vaginal removal techniques in patients with MRUO.

## 2 MATERIALS AND METHODS

After receiving Institutional Review Board (IRB) approval (IRB number: E‐83045809‐903.99‐755883), we conducted a retrospective review of those women who underwent unilateral J‐cut division, partial and subtotal vaginal removal surgeries for MRUO at our six university hospitals between 2002 and 2022. Patients with inadequate data, mesh exposure and extrusion (vagina, urethra and bladder), neurogenic bladder and concomitant surgery were excluded. The patient review included demographics, a detailed medical history and proforma with details of lower urinary tract symptoms (LUTS), physical and urodynamic findings, detailed surgical reports and follow‐up data. During the follow‐up period, the patients' satisfaction status was evaluated by the yes or no question ‘were you satisfied with the sling revision surgery?’

We defined MRUO as a persistently raised post‐void residual, troublesome voiding LUTS such as prolonged and positional voiding, hesitancy and straining, difficulty emptying to completion, the need for indwelling and clean intermittent self‐catheterization (CIC), as well as the urodynamic female bladder outlet obstruction (BOO) findings arising after mid‐urethral sling surgery (MSS).^20^ De novo SUI was defined as the recurrence of the symptom and/or urodynamic finding of SUI after SRS.

**FIGURE 1:**
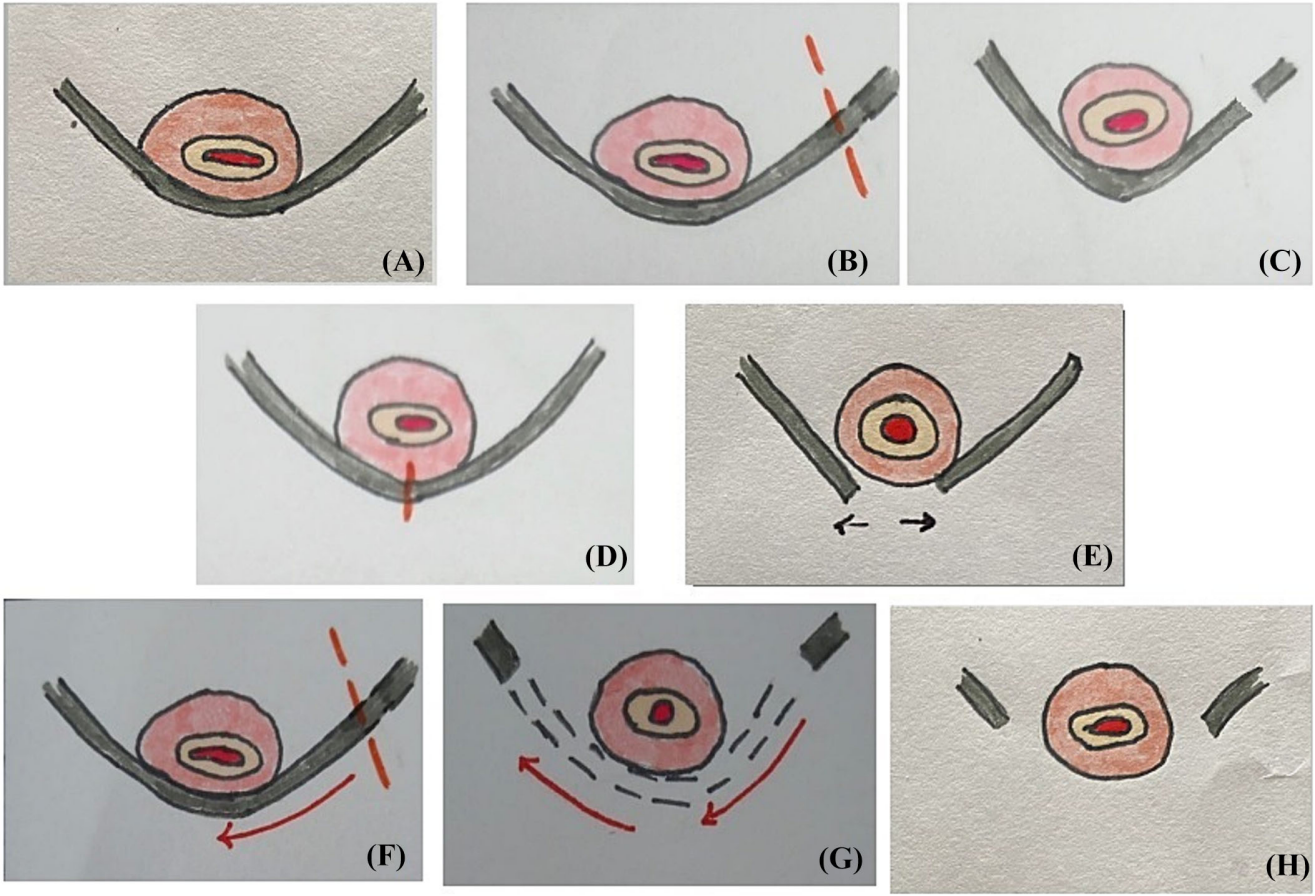
(A) Sling‐related obstruction, (B) unilateral sling division, (C) unilateral ‘J‐cut’ sling division, (D) midline sling dissection and division, (E) partial vaginal removal, (F) unilateral sling dissection and division, (G) peeling the sling from underneath the urethra towards the contralateral ischiopubic ramus, (H) subtotal vaginal removal.

Three different SRS approaches were used (unilateral J‐cut division [Figure 1A–C], described by Kasturi et al., partial [Figure 1D,E] and subtotal vaginal removal [Figure 1F–H] techniques).^14^ The partial vaginal removal technique consisted of midline sling dissection and division, and after that, peeling and removing each end of the sling 1.5 cm long from underneath the urethra (Figure 1D,E). The subtotal vaginal removal technique consisted of unilateral sling dissection and division, and thereafter, peeling and removing of the sling from underneath the urethra towards the contralateral ischiopubic ramus^21^ (Figure 1F–H). The choice of the technique was at the discretion of the operating surgeon.

The Clavien–Dindo system was used on a scale of 1–5 to grade the complications.^22^

Functional (obstruction relieving) outcomes, complications (recurrence of SUI), the patient satisfaction status and the duration of SRS were compared between the three groups.

### 2.1 Statistical analysis

Statistical analysis of all data was evaluated using SPSS Statistics (Version 21.0, IBM Corp) package programme. All numerical values were expressed as median (interquartile range [IQR] 25–75). Categorical data were expressed in terms of frequency and percentage. The suitability of the quantitative data to normal and abnormal distribution was evaluated using the Kolmogorov–Smirnov normality test. The Kruskal–Wallis test was used to compare the quantitative data that did not fit the normal distribution. χ^2^ and Fisher's exact tests were used in the analysis of categorical variables. Wilcoxon test was used to compare the quantitative values of the dependent groups before and after surgery that did not fit the normal distribution. The statistical significance limit of all evaluations was accepted as *p* < 0.05.

## 3 RESULTS

**FIGURE 2:**
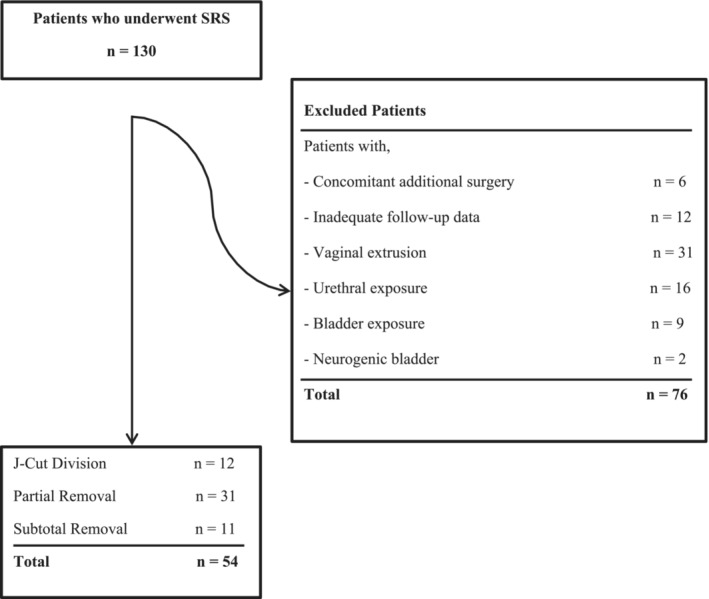
Flow chart of inclusion and exclusion criteria.

SRS was performed in 130 female patients with mesh‐related complications after MSS. After exclusion criteria, 54 women with a median age of 57 (47–64) y/o who underwent unilateral J‐cut division, partial and subtotal vaginal removal surgery for MRUO were eligible for the study (Figure 2). The overall satisfaction rate was 84.6% at a median follow‐up of 48 (17–96) months.

Unilateral J‐cut division, partial and subtotal vaginal removal surgeries were performed in 12, 31 and 11 patients with a median duration of surgery of 30 (25–34), 40 (35–56) and 60 (60–70) minutes, respectively (*p* = 0.001).

Patient characteristics (age, main presenting symptom, duration, additional LUTS, medical treatment and type of MSS) before SRS and duration of follow‐up of each group were shown in Table 1. Voiding difficulty and urinary incontinence were the main presenting symptoms in 49 patients (90.7%).

**TABLE 1:** Patient characteristics.

Variables	Groups			
	J‐Cut division (*n* = 12)	Partial removal (*n* = 31)	Subtotal removal (*n* = 11)	*p* value
Age (years)	56 (45–62)	56 (46–66)	61 (52–66)	0.557
Main presenting symptoms (overall, %)				
Incontinence	5 (41.7)	9 (29)	5 (45.5)	
Voiding difficulty	6 (50)	18 (58.1)	6 (54.5)	
Overactive bladder	1 (8.3)	0 (0)	0 (0)	
Others	0 (0)	4 (12.9)	0 (0)	
Duration of main presenting symptom (months)	10 (3–39)	12 (3–30)	24 (24–96)	0.011
Additional LUTS (overall, %)				
Storage LUTS	10 (83.3)	28 (90.3)	11 (100)	
Voiding LUTS	6 (50)	27 (87.1)	10 (90.9)	
Mixed LUTS	6 (50)	24 (77.4)	10 (90.9)	
Medical treatment before SRS (overall, %)				0.178
None	7 (58.3)	20 (64.5)	10 (90.9)	
Anticholinergic/mirabegron	5 (41.7)	11 (35.5)	1 (9.1)	
Type of MUS surgery (overall, %)				0.105
TOT	6 (50)	25 (80.6)	7 (63.6)	
TVT	6 (50)	6 (19.4)	4 (36.4)	
Interval between sling placement and SRS (months)	12 (7–51)	12 (6–48)	24 (6–60)	0.702
Duration of follow‐up (months)	44 (12–120)	37 (15–93)	72 (36–99)	0.619

*Note*: Data were expressed as median (interquartile range) or number (percentage) whenever appropriate.

Abbreviations: LUTS, lower urinary tract symptoms; MUS, midurethral sling; SRS, sling revision surgery; TOT, transobturator tape; TVT, transvaginal tape.

The comparison of the functional (obstruction relieving) outcomes, complications (recurrence of SUI), the patient satisfaction status and the duration of SRS were shown in Table 2. A formal multichannel urodynamic study was performed in 49 women (90.7%) before SRS. BOO was determined in 75.5% of these patients based on the urodynamic female BOO criteria.^20^ BOO was not found in any patient with post‐operative urodynamic investigation (Table 2).

**TABLE 2:** Comparison of functional outcomes, duration of SRS, de‐novo SUI, persistent UUI and satisfaction status between groups.

Variables	Groups			
	J‐Cut division (*n* = 12)	Partial removal (*n* = 31)	Subtotal removal (*n* = 11)	*p* value
Preoperative urodynamic BOO^a^ (overall, %)	6/9 (66.6)	22/30 (79.2)	9/10 (90)	
Postoperative urodynamic BOO^b^ (overall, %)	0/1 (0)	0/12 (0)	0/11 (0)	
Preoperative voiding status (overall, %)				
Spontaneous	9 (75)	29 (93.5)	9 (81.8)	
CIC	2 (16,7)	1 (3.2)	1 (9.1)	
Indwelling catheter	1 (8.3)	1 (3.2)	1 (9.1)	
Postoperatively voiding status (overall, %)				
Spontaneous	12 (100)	31 (100)	11 (100)	
CIC	0 (0)	0 (0)	0 (0)	
Indwelling catheter	0 (0)	0 (0)	0 (0)	
Preoperatively Qmax, mL/s	8 (0–11)	9 (7–12)	5 (1–9.5)	0.099
Postoperatively Qmax, mL/s	21 (19–28)	19 (13–21.5)	18 (17–23)	0.245
*p* value	0.018	0.001	0.012	
Preoperatively post‐void residual urine volume, mL	200 (150–288)	140 (58–220)	275 (112.5–375)	0.067
Postperatively post‐void residual urine volume, mL	10 (0–50)	60 (30–105)	30 (10–50)	0.01
*p* value	0.012	0.002	0.008	
Presence of de‐novo SUI (overall, %)	1/10 (10)	11/25 (44)	6/10 (60)	0.007
Presence of persistent UUI (overall, %)	4/9 (44.4)	3/11 (27.2)	1/8(12.5)	0.444
Duration of surgery (minutes)	30 (25–34)	40 (35–56)	60 (60–70)	0.001
Satisfaction after SRS^c^ (overall, %)				0.083
Yes	10 (90.9)	23 (76.7)	11 (100)	
No	1 (9.1)	7 (23.3)	0 (0)	

*Note*: Data were expressed as median (interquartile range) or number (percentage) whenever appropriate. De‐novo SUI rates were calculated only for patients without preoperative SUI.

Abbreviations: BOO, bladder outlet obstruction; CIC, clean intermittent self‐catheterization; SRS, sling revision surgery; SUI, stress urinary incontinence; UUI, urge urinary incontinence.

^a^ Multichannel urodynamics was performed in 49 patients.

^b^ Multichannel urodynamics was performed on 24 patients.

^c^ The question ‘Were you satisfied with the sling revision surgery?’ had been asked to the patients during the follow‐up period.

Seven patients were either performing CIC or had indwelling catheters because of varying degrees of urinary retention before SRS, while all of these patients voided spontaneously after the surgery (Table 2).

A statistically significant increase in median maximum free urine flow rate and decrease in median post‐void residual urine volume were found after SRS in the three groups, while de novo SUI developed in 10%, 44% and 60% of the patients in the unilateral J‐cut division, partial and subtotal vaginal removal groups, respectively (*p* = 0.007) (Table 2). The rates of persistent urgency urinary incontinence (UUI) in the unilateral J‐cut division, partial and subtotal vaginal removal groups were 44.4% (4 out of 9 patients), 27.2% (3 out of 11 patients) and 12.5% (1 out of 8 patients), respectively. However, there was no statistically significant difference between the groups in this sample size (Table 2).

Two patients in the partial removal group with midline division had an intraoperative urethral injury (Grade IIIb) and had an immediate surgical correction. These two patients had longer (7 days) urethral catheterization post‐operatively with a satisfactory outcome. The complication of de novo SUI occurred in 18 patients, of whom two had MSS (Grade IIIb), and the remaining patients had or were advised to use the medical treatment and/or pelvic floor physiotherapy (Grade I).

Patient satisfaction status evaluated by yes or no questions in unilateral J‐cut division, partial and subtotal vaginal groups revealed that 90.9%, 76.7% and 100% of the patients were satisfied with SRS, respectively. The overall satisfaction rate was 84.6% (Table 2).

## 4 DISCUSSION

Evidence on the outcomes of SRS is continuously growing as studies focusing on this subject have been reported recently. However, most published studies have been too heterogenous, usually consisting of female patients with every kind of mesh‐related complication.^3^, ^4^, ^9^, ^10^, ^23^ It is logical to perform total mesh removal in patients with pain, infectious exposure and extrusion complications. On the other hand, total mesh removal is a complex and time‐consuming surgery and usually not being used for female patients with MRUO. Although there is a tendency to perform the simple division technique with a midline or lateral approach in patients with MRUO, partial and subtotal vaginal removal techniques are still being used in this patient population.^6^, ^7^, ^8^, ^12^, ^13^, ^14^, ^15^, ^16^, ^17^, ^18^, ^19^ Simple sling division by a midline incision, and partial/subtotal vaginal removal surgery peeling the sling underneath the urethra may compromise support under the urethra and potentially increase the risk of SUI recurrence.^14^ We need unique recommendations to choose the ideal surgical technique for managing female patients with MRUO. The present study aimed to provide evidence on this subject, and to our knowledge, is the first study comparing the well‐defined unilateral J‐cut division with partial and subtotal vaginal removal techniques in a homogeneous group of female patients with MRUO.

Two retrospective studies compared the outcomes of the division and partial removal surgery in 238 patients with MSS‐related voiding dysfunction and concluded that both techniques successfully relieved obstruction.^10^, ^19^ The objective functional outcome parameters were not presented in both of these studies. The present study with long‐term follow‐up data compared the three different techniques on the basis of obstruction relief (by means of post‐void residual urine measurement and appropriate urodynamic methods), de novo SUI and persistent UUI. Long‐term follow‐up data demonstrated that the unilateral J‐cut division technique with a statistically significant shorter duration of surgery time and lower risk of de novo SUI was as effective as the partial and subtotal removal techniques in relieving MRUO and could be recommended as the procedure of choice in the homogeneous group of patients with MRUO. This recommendation should be supported with future well‐designed comparative studies with a larger number of patients.

Three studies compared the division and partial removal techniques in 340 patients with MSS complications based on de novo SUI, and two of these studies found a lower risk of recurrent SUI in simple division technique while the remaining study did not.^6^, ^10^, ^19^ The present study demonstrated that the risk of de novo SUI in patients with unilateral J‐cut division surgery was significantly lower than in those with partial and subtotal vaginal removal surgery.^6^, ^7^, ^9^, ^10^

Persistence of storage symptoms and UUI after SRS may often be troublesome. In the present series, the rates of persistent UUI in the unilateral J‐cut division, partial and subtotal vaginal removal groups were 44% (4 out of 9 patients), 27.2% (3 out of 11 patients) and 12.5% (1 out of 8 patients), respectively. However, there was no statistically significant difference between the groups in this sample size. Series with a larger number of patients would be more informative on this issue.

Kasturi et al., who described a novel unilateral J‐cut division technique, commented that the sling division in the midline might compromise support under the urethra and potentially increase the risk of de novo SUI.^14^ Two centres of the present multicentric study used the unilateral J‐cut division technique in 12 patients, and the risk of de novo SUI was found to be significantly lower in these patients than in the ones who had partial and subtotal vaginal removal surgery. Furthermore, partial removal techniques using midline incision compared with the unilateral division technique also carry more potential risk of urethral injury because the dissection procedure in these techniques is very close to the urethra. Furthermore, translabial ultrasound studies show a shorter distance of the mesh to the urethral lumen in women with obstruction after midurethral sling (MUS) surgery increasing the potential risk of urethral injury in SRS with midline incisions.^24^ In the present study, the partial vaginal removal technique with midline incision used in three centres consisted of a mesh‐releasing procedure very close to the urethra in the midline and two urethral injuries occurred. No urethral injury occurred in the patients who had either the unilateral J‐cut division or the subtotal vaginal removal surgery in which the sling was divided laterally. Future studies with a larger number of patients comparing the unilateral and midline division techniques in patients with MRUO are needed to determine the most favourable technique regarding the urethral injury complication.

The terminology of the SRS techniques such as the division, partial, subtotal and total removal of mesh must be clearly defined and standardized to compare the efficacy and complications of the different techniques of SRS.^4^, ^25^, ^26^ In the present study, the mesh division involved only cutting the mesh unilaterally without removing any part of it, while the partial and subtotal vaginal removal consisted of varying degrees of segmental removal of the mesh with a vaginal midline or lateral approaches.

Our study, with its retrospective design, is subject to some limitations, including inadequate data collection in a non‐standardized manner. We excluded the patients with inadequate data, which diminished the effect of these limitations to some extent. Because we chose to conduct this study in a homogeneous cohort of patients with MRUO, we excluded many patients with other MSS‐related complications, resulting in a relatively small sample size, which was another limitation of this study. The strengths of our study included the comparison of well‐defined unilateral J‐cut division, partial and subtotal vaginal removal techniques in a homogeneous group of patients with MRUO, with functional outcome data and long‐term follow‐up. Furthermore, the duration of surgery time and the complication of de‐novo SUI rates of the three different techniques with adequate data were compared.

## 5 CONCLUSIONS

The unilateral J‐cut division technique was as effective as the partial and subtotal vaginal removal techniques in relieving MRUO with a shorter duration of surgery time (*p* = 0.001) and lower risk of de novo SUI (*p* = 0.007). Comparative studies with a larger number of patients are needed.

## AUTHOR CONTRIBUTIONS

The authors confirm their contributions to the paper as follows: study conception and design: Bülent Çetinel; data collection: Ömer Gülpınar, Aydın Yenilmez, Adnan Şimşir, Alkan Çubuk, Gökhan Temeltaş, Göktuğ Kalender and Elif Altınay Kırlı; analysis and interpretation of results: Günay Can and Göktuğ Kalender; draft manuscript preparation: Bülent Çetinel, Elif Altınay Kırlı and Göktuğ Kalender. All authors reviewed the results and approved the final version of the manuscript.

## CONFLICT OF INTEREST STATEMENT

There is no conflict of interest.
